# Supplementary material for: Venomix: a simple bioinformatic pipeline for identifying and characterizing toxin gene candidates from transcriptomic data
Source: PeerJ. 2018 Jul 31;6:e5361. doi: 10.7717/peerj.5361 (PMC6074769; doi:10.7717/peerj.5361)
Supplement: Supplemental Information 3 [file peerj-06-5361-s003.gz › FinalOutput_E-6/Kunitz-type_conkunitzin-S1_2/finaltree.pdf]

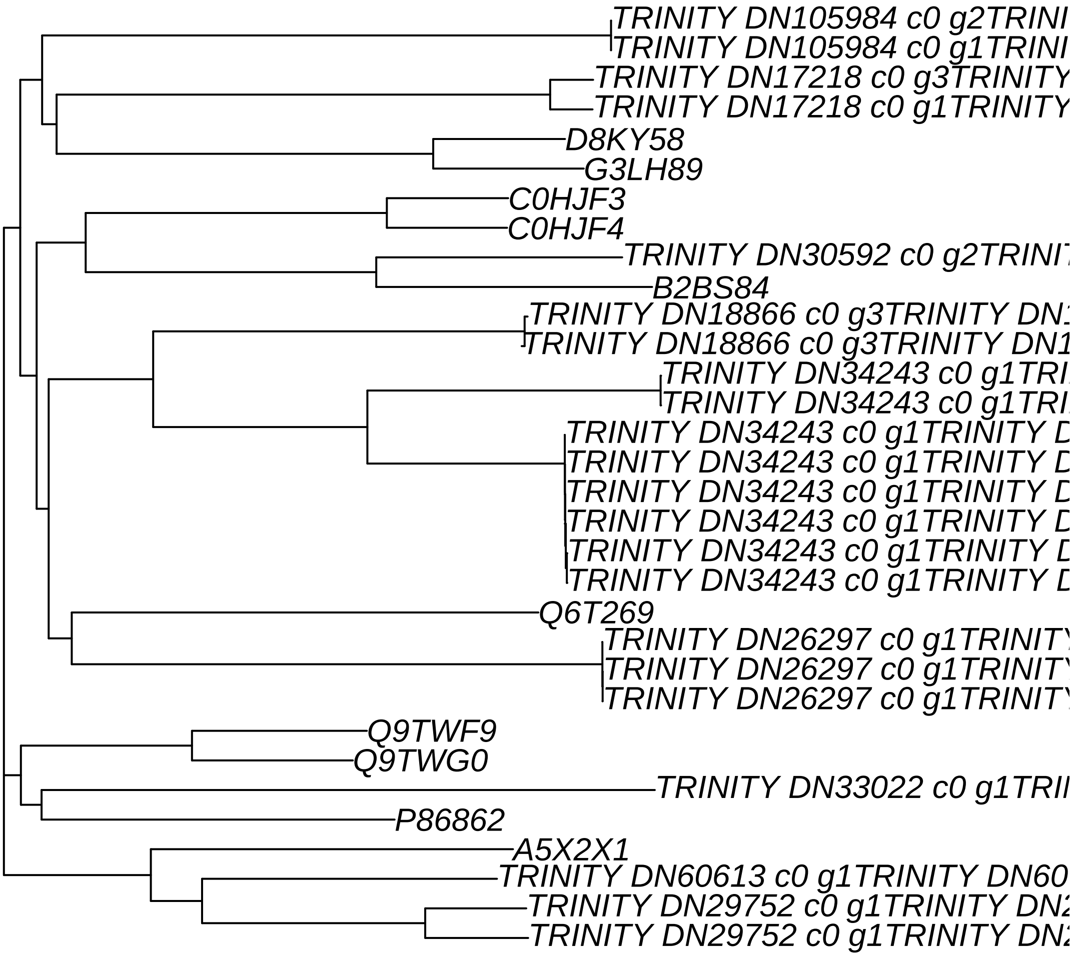
TRINITY DN105984 c0 g2TRINI
TRINITY DN105984 c0 g1TRINI
TRINITY DN17218 c0 g3TRINITY
TRINITY DN17218 c0 g1TRINITY
D8KY58
G3LH89
C0HJF3
C0HJF4
TRINITY DN30592 c0 g2TRINIT
B2BS84
TRINITY DN18866 c0 g3TRINITY DN1
TRINITY DN18866 c0 g3TRINITY DN1
TRINITY DN34243 c0 g1TRI
TRINITY DN34243 c0 g1TRI
TRINITY DN34243 c0 g1TRINITY D
TRINITY DN34243 c0 g1TRINITY D
TRINITY DN34243 c0 g1TRINITY D
TRINITY DN34243 c0 g1TRINITY D
TRINITY DN34243 c0 g1TRINITY D
TRINITY DN34243 c0 g1TRINITY D
Q6T269
TRINITY DN26297 c0 g1TRINITY
TRINITY DN26297 c0 g1TRINITY
TRINITY DN26297 c0 g1TRINITY
Q9TWF9
Q9TWG0
TRINITY DN33022 c0 g1TRI
P86862
A5X2X1
TRINITY DN60613 c0 g1TRINITY DN60
TRINITY DN29752 c0 g1TRINITY DN2
TRINITY DN29752 c0 g1TRINITY DN2
